# Prevalence and risk factors of drug-related hospitalizations in multimorbid patients admitted to an internal medicine ward

**DOI:** 10.1371/journal.pone.0220071

**Published:** 2019-07-22

**Authors:** Marianne Lea, Morten Mowe, Liv Mathiesen, Kristin Kvernrød, Eva Skovlund, Espen Molden

**Affiliations:** 1 Department of Pharmaceutical Services, Oslo Hospital Pharmacy, Hospital Pharmacies Enterprise, South Eastern Norway, Oslo, Norway; 2 General Internal Medicine Ward, the Medical Clinic, Oslo University Hospital, Oslo, Norway; 3 Faculty of Medicine, University of Oslo, Oslo, Norway; 4 Hospital Pharmacies Enterprise, South Eastern Norway, Oslo, Norway; 5 Department of Pharmacy, Section for Pharmacology and Pharmaceutical Biosciences, University of Oslo, Oslo, Norway; 6 Department of Public Health and Nursing, Faculty of Medicine and Health Sciences, Norwegian University of Science and Technology, NTNU, Trondheim, Norway; 7 Center for Psychopharmacology, Diakonhjemmet Hospital, Oslo, Norway; Newcastle University, UNITED KINGDOM

## Abstract

**Background:**

Knowledge of risk factors for drug-related hospitalizations (DRHs) is limited.

**Aim:**

To examine the prevalence of DRHs and the relationships between DRHs and various variables in multimorbid patients admitted to an internal medicine ward.

**Methods:**

Multimorbid patients ≥ 18 years, using minimum of four regular drugs from minimum two therapeutic classes, were included from the Internal Medicine ward, Oslo University Hospital, Norway, from August 2014 to March 2016. Clinical pharmacists prospectively conducted medicines reconciliations and reviews to reveal drug-related problems (DRPs). Blinded for identified DRPs, an interdisciplinary group retrospectively made comprehensive, clinical assessments of each patient case to classify hospitalizations as drug-related (DRH) or non-drug-related (non-DRH). Age, sex distribution, Charlson Comorbidity Index (CCI), renal function, aberrant genotype frequencies, body-mass index, number of drugs, proportion of patients which received assistance for drug administration from the home care service, and/or through multidose-dispensed drugs, and occurrence of specific DRP subgroups, were compared separately between patients with DRHs versus non-DRHs, followed by multiple logistic regression analysis.

**Results:**

Hospitalizations were classified as drug-related in 155 of the 404 included patients (38%). Factors significantly associated with DRHs were occurrence of adverse effect DRPs (adjusted odds ratio (OR) 3.3, 95% confidence interval (CI) 1.4–8.0), adherence issues (OR 2.9, 1.1–7.2), home care (OR 1.9, 1.1–3.5), drug monitoring DRPs (OR 1.9, 1.2–3.0), and CCI score ≥6 (OR 0.33, 0.14–0.77). Frequencies of aberrant genotypes did not differ between the patient groups, but in 41 patients with DRHs (26.5%), gene-drug interactions influenced the assessments of DRHs.

**Conclusion:**

DRHs are prevalent in multimorbid patients with adverse effect DRPs and adherence issues as the most important risk factors.

## Introduction

Many previous studies have investigated the occurrence of drug-related hospitalizations (DRHs) in different patient populations [1–5]. A systematic review representing various patient groups reported that around 10% of all hospitalizations are drug-related [6]. In older adults, the reported prevalence of DRHs is higher, comprising around 30% of all hospitalizations [7, 8].

Preventing hospitalizations is important both for the benefit of the patients and society. A high proportion of DRHs are preventable [1–3, 6, 7, 9, 10], but effective prevention requires knowledge of important risk factors. The causality of DRHs may be complex and involve many predisposing factors, such as patient characteristics, disease state, living situation, and drug-related problems (DRPs) [1–3, 6, 7, 11–15]. A DRP is defined as an event or circumstance that actually or potentially interferes with desired health outcomes [16], which can manifest as drug-related morbidity or death if no action is taken [17]. Typical DRPs comprise suspected adverse effects, unnecessary drugs, adherence issues and drug-drug interactions (DDIs). Moreover, pharmacogenetic variability is a potential source of DRPs [18], which has not previously been investigated in relation to risk of DRHs.

Multimorbid patients represent a group with a high risk of DRPs [17]. This is a heterogeneous patient group, but a common feature is increased health care utilization, including frequent hospitalizations, and multiple drug treatments [19–23]. The population of multimorbid patients is growing due to steadily improving health care and increasing life expectancy [24, 25]. These patients are often admitted to internal medicine wards due to the complex nature of their disease state. They, therefore, comprise a resource-demanding patient group, where prevention of hospitalizations is crucial to reduce social costs and improve patient health.

Several studies have previously investigated the prevalence and risk factors for DRHs in various patient populations, e.g. cancer patients, geriatric patients and older patients with dementia [4, 8, 26]. Advanced age and polypharmacy are the main risk factors for DRHs identified in most studies [6, 27, 28]. However, to the best of our knowledge, no studies have investigated multimorbid patients with respect to prevalence and risk factors for DRHs. The aim of the present study was therefore to examine the prevalence of DRHs and the relationships between DRHs and various variables in multimorbid patients admitted to an internal medicine ward.

## Materials and methods

### Study design and setting

This observational study, approved by the Regional Committee for Medical and Health Research Ethics (2014/704/REK south-eastern D) and the Privacy Ombudsman, was conducted at an internal medicine ward of Oslo University hospital (Ullevaal location), Norway, from August 2014 to March 2016. The current study is based on observational data collected during the inclusion of patients to a randomized controlled trial (RCT) studying the effect of a pharmacist intervention on readmissions, ClinicalTrials.gov Identifier: NCT02336113. Baseline data at inclusion was used to assess drug-related hospitalizations. Patients were considered for inclusion by clinical pharmacists Monday to Friday during regular daytime working hours until a target number of 400 patients, based on the power calculation of the RCT, were enrolled. Eligible patients were prospectively invited and enrolled in the study following written informed consent.

**Fig 1:**
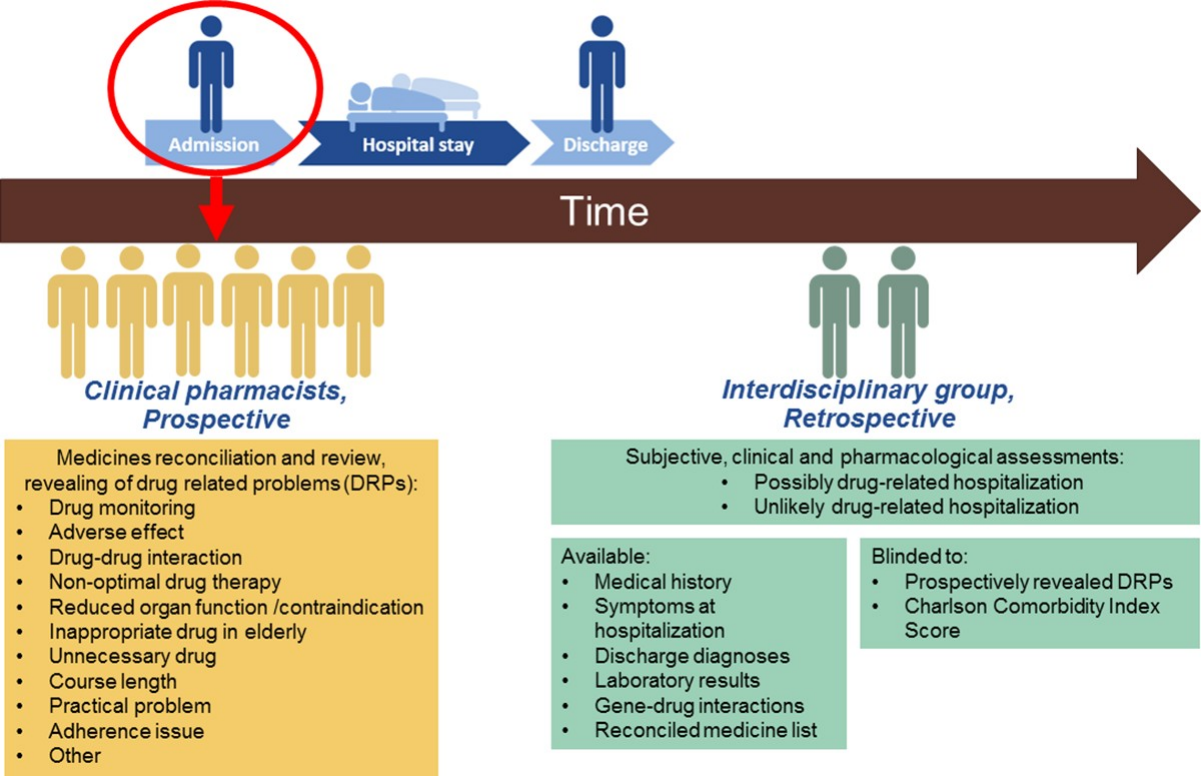
Illustration of the outline of the study.

Fig 1 illustrates the outline of the study with the various steps and processes performed after patient inclusion. During the inclusion period, six experienced clinical pharmacists, all with a master degree in clinical pharmacy and had undergone a thorough, standardized course in IMM, prospectively performed medicine reviews and identified DRPs at the time of admission, based on the reconciled drug list as described below. As the pharmacists worked daytime shifts, more than one pharmacist was often involved in an individual participant`s medicines reconciliations and/or reviews. Information about sex, age, body-mass index (BMI) and living situation. If the patient received assistance with drug administration from home care, in the following text referred to as ‘home care’, and/or through multidose-dispensed drugs, this was also registered. Multidose-dispensed drugs mean that the individual patient’s drugs (tablets or capsules) were prepacked by a dispensing pharmacy in small, labeled plastic bags; each bag contains all drugs prescribed to be administered at the same time.

In addition, blood samples were collected for biochemical measurements and pharmacogenetic analyses of drug-metabolizing cytochrome P450 (CYP) enzymes and the transporter mediating uptake of statins from the blood into the liver (OAPTP1B1). Glomerular filtration rate (GFR) was calculated using the Cockcroft-Gault formula [29], except for obese patients (BMI > 30) where the Salazar-Corcoran formula was used [30]. An experienced senior physician retrospectively collected information of diagnoses from the medical records to calculate the Charlson Comorbidity Index (CCI) score of each patient [31].

### Inclusion and exclusion criteria

Inclusion criteria were: acute admission, age ≥ 18 years and use of at least four regular drugs from at least two Anatomical Therapeutic Chemical (ATC) groups [32] at 1st level, at admission. The latter was a surrogate for multimorbidity, defined as the presence of minimum two conditions, a commonly used definition [33]. Exclusion criteria were i) terminally ill, ii) isolated due to severe infections, or iii) unable to communicate in Norwegian or English in absence of a translator. Patients readmitted during the study period were not invited for ‘a second’ inclusion.

### Prospective medicine reconciliation and review

A Norwegian translation of the Integrated Medicines Management (IMM) model [34], adapted to the Norwegian setting, was used as the method for the systematic medicines reconciliations and reviews. Medicines reconciliation was performed by clinical pharmacists according to a structured IMM interview form, which included questions to assess patient adherence. Based on the reconciled medicine list, the clinical pharmacists performed systematic medicines reviews and identified DRPs at the time of admission. These medicines reviews were defined as *advanced* reviews, according to the PCNE classification [35]. The medicine reviews only included drugs used prior to admission and not drugs initiated during transport to or following hospital admission. The pharmacists had access to the patient’s medical history and laboratory results (up to and including admission time) when performing medicine reviews.

A DRP was defined according to the Pharmaceutical Care Network Europe (PCNE) as ‘an event or circumstance involving drug therapy that actually or potentially interferes with desired health outcomes’ [16]. As IMM was used by the clinical pharmacists to conduct medicine reviews, the identified DRPs were classified in the following subgroups according to the IMM procedure; ‘drug monitoring’, ‘adverse effect’, ‘drug-drug-interaction’, ‘non-optimal drug therapy’, ‘reduced organ function / contraindication’, ‘inappropriate drug in elderly’, ‘unnecessary drug’, ‘course length’, ‘practical problem’, ‘adherence issue’ and ‘other’. Each DRP subgroup is described in detail in S1 Table. When information obtained during medicines reconciliation led to the identification of a DRP, this was also recorded.

### Pharmacogenetic analyses

Blood samples drawn from the included patients were used for pharmacogenetic analyses at Diakonhjemmet Hospital, Oslo, Norway. The analyses were conducted retrospectively due to time constraints and therefore not included as a basis for the medicine reviews. For all included patients, variant allele analyses of *CYP2D6*, *CYP2C19*, *CYP2C9*, *CYP3A5* and *SLCO1B1* (encoding OATP1B1; uptake transporter of statins into the liver) were performed. For warfarin-treated patients, *VKORC1* genotyping was also conducted. An overview of the variant alleles (mutations) of the various pharmacogenes included in the genotyping panels, and the respective genotype-predicted aberrant phenotypes, is provided in S2 Table. Briefly, for CYP2D6 and CYP2C19, the most relevant polymorphic enzymes involved in drug metabolism, homozygous carriers of non-coding (*null*) alleles were defined as ‘poor metabolizers’ (PMs) of the respective enzymes, while heterozygous carriers of *null* alleles and homozygous carriers of reduced-function alleles were defined as ‘intermediate metabolizers’ (IMs). Patients carrying three or more functional *CYP2D6* gene copies were classified as CYP2D6 ‘ultrarapid metabolizers’ (UMs), while patients carrying CYP2C19*17 were classified as CYP2C19 UMs.

### Identification of gene-drug interactions (GDIs)

Retrospectively, the authors EM and ML identified *gene-drug interactions* (GDIs) by assessing the reconciled drug list against the respective patients`genotype results to identify drugs being substrates of the respective polymorphic enzymes or transporters. Assessments were restricted to aberrant phenotypes, as defined in S2 Table. The concept of defining a gene-drug interaction (GDI) was that a patient’s variant genotype likely determined an at least 50% change in the plasma concentration/exposure of the affected drug. As there is currently no gene-drug interaction databases available, each of the cases was assessed by manual reviews with author EM as the responsible person, who is head of research at the Center for Psychopharmacology, where genotyping is performed as a clinical service on a routine basis. In the assessments, a conservative approach was applied concerning i) the variant genotype’s effect on function/phenotype of the enzyme or transporter (expected reduction or increased in activity score by a factor of 2 or more), and ii) the estimated relative involvement of the enzyme or transporter in the overall clearance of the drug (expected fraction of clearance ≥1/3). The presumed clinical relevance of the identified GDIs was included in the assessments of drug-related hospitalizations.

### Assessment of drug-related hospitalization (DRH)

After patient inclusion, a senior geriatrician/internal medicine physician (author MM) and a pharmacologist (author EM) made comprehensive, clinical and pharmacological assessments for each individual patient as to whether the hospitalization was *possibly* drug-related (classified as DRH) or *unlikely* to be drug-related (classified as non-DRH). This method for hospitalization assessment with only two categories, i.e. ‘possibly’ or ‘unlikely’ drug-related, was applied due to the limitations of using scoring tools to grade the probability of DRHs in this population, where patients are characterized by complex symptoms and drug treatments.

Prior to the assessments, the authors MM and EM received case report forms (CRFs) prefilled by the clinical pharmacist who organized the study (author ML). The following information was included in the CRFs: sex, age, brief medical history, symptoms at hospitalization, laboratory results, reconciled drug list at hospitalization, discharge diagnoses, and results from the pharmacogenetic analyses. MM and EM were blinded to the CCI score and the DRPs prospectively identified by the study pharmacists, and they had no training or knowledge of identification of DRPs according to IMM. The main principle during the DRH assessments was whether the diversity of symptoms, laboratory values and/or current admission reasons mentioned in the medical record were *possibly* or *unlikely* explained by the patient`s drug use or lack of drug use. The assessments were made in physical meetings, where EM and MM together discussed each case thoroughly (15–20 minutes per patient) until agreement was reached, to classify hospitalizations as *possibly* or *unlikely* drug-related. Drugs involved in possible DRHs, and GDIs that influenced the DRH assessments, were systematically registered.

### Statistics

All registered patient variables, i.e. age, sex, number of prescribed drugs, living situation, use of home care, multidose-dispensed drugs, BMI, GFR, CCI score, genotype frequencies and occurrence of specific DRP subgroups, were initially compared between patients with DRHs versus non-DRHs using chi-square test for proportions and Mann-Whitney test for continuous variables. Variables with p values < 0.2 in the simple comparisons were included in the subsequent multiple logistic regression analysis. Backward elimination of non-significant variables was performed, and the final model was restricted to include explanatory variables with p values < 0.05. Adjusted odds ratios (ORs) with 95% confidence intervals (CIs) were reported. Excluded variables were introduced one by one to the final model to verify that they did not significantly affect the effect of the variables that remained in the model.

IBM SPSS Software version 25.0 (IBM Corp. NY), was used for all statistical analyses. P values < 0.05 were defined as statistically significant.

## Results

During the study period, 2174 patients were admitted to the internal medicine ward and 1769 patients (81%) were considered for inclusion. Of these, 913 patients did not meet the predefined inclusion criteria and 258 patients were not asked to participate for practical reasons. Among the remaining 598 patients, 175 (29%) declined the invitation to participate in the study (permission to register reasons for declining not obtained). After excluding patients due to i) medicines reconciliation revealed use of less than four regular drugs (n = 3), ii) erroneous reinclusion (n = 1), iii) withdrawal of consent (n = 8), and iv) acute events preventing data collection (in-hospital death; n = 3, transfer to other wards; n = 3, rapid discharge; n = 1), a total of 404 participants were enrolled in the study.

The median age of the included patients was 79.4 years (range 23.1–96.4) and 216 patients (54%) were females. Most patients (n = 373, 92%) were living at home prior to hospitalization, and the majority (n = 283, 70%) administered the drugs themselves. The median number of regular and as needed drugs in the study population was 8 (range 4–19) and 2 (range 0–11) and the median number of diagnoses was 7 (range 2–17), emphasizing the multimorbidity of the study participants. The median CCI score was 3 (range 0–12). The five most frequent reasons for hospitalization described in the medical admission record were infections (n = 72), breathlessness (n = 44), heart failure exacerbation (n = 37), chest pain (n = 35) and general functional impairment/failure (n = 27). During the hospital stay, 11 of the 404 included participants died. We did not have permission to register the causes of death or to assess these as drug-related or not.

In the medication reviews, the study pharmacists identified a total number of 5527 DRPs, whereof 950 (17%) were identified as a result of completing medicine lists during medicines reconciliation. The median number of DRPs per patient was 13 (range 3–42), and 315 patients (78%) had a minimum of one DRP identified based on information revealed during the medicines reconciliation.

After comprehensive clinical and pharmacological assessments using information about patient characteristics, disease status and history, laboratory results, reconciled medicine lists, discharge diagnoses and pharmacogenetic profiles of all included patients, authors MM and EM retrospectively classified hospitalizations as possibly drug-related in 155 patients (38%). S3 Table shows the drugs most frequently involved in the underlying causes of hospitalization in the cases assessed as DRHs. Metoprolol was the drug most frequently suspected to be related to DRHs, i.e. in as many as 39 of the cases (25%). The second most frequent drug suspected to be related to hospitalization was bumetanide (21 cases), followed by zopiclone (15 cases) and insulin or insulin analogues (13 cases).

In 287 of the patients (71%), at least one gene-drug interaction (GDI) was identified (total number of GDIs 538). One or more GDIs were found to be of relevance for the DRH assessments in 41 of the patients (26.5%). In five patients, GDIs were decisive for classifying hospitalizations as possibly drug-related. S4 Table shows frequencies of the various GDIs identified in the patient population, as well as those relevant for DRH assessments. The most frequently involved agents in GDIs were metoprolol, comprising 125 of the 538 GDIs (23%), and warfarin, comprising 60 of the 538 GDIs (11%). Proton pump inhibitors, statins, and opioids were also commonly involved in GDIs, with 84, 81 and 74 identified cases.

For 9 patients the relationship between drug treatment and hospitalization was not assessed due to insufficient information in the medical record. These patients were excluded in the subsequent comparative analyses of patients with *possibly* DRHs versus *unlikely* DRHs, in the following text described as ‘DRHs’ (155 patients) versus ‘non-DRHs’ (240 patients).

In Table 1, patients with DRHs and non-DRHs are compared with respect to age, sex, number of prescribed drugs, living situation, home care, multidose-dispensed drugs, BMI, GFR, CCI score and frequencies of variant genotypes encoding reduced/absent enzyme or transporter activities. The proportion receiving multidose-dispensed drugs was the only of these variables being significantly different between the cases of DRHs versus non-DRHs in the unadjusted analysis, i.e. 30% versus 20% (p = 0.035). In addition, home care, CCI score, BMI and number of prescribed drugs had p values < 0.2 for comparisons between DRHs and non-DRHs, and were included as candidate variables in the subsequent multiple logistic regression analysis.

**Table 1:** Comparisons of characteristics in patients with drug-related hospitalizations (DRHs) versus non-drug-related hospitalizations (non-DRHs) in the study population (n = 395^a^).

Characteristic	DRHs (n = 155)	Non-DRHs (n = 240)	p value
Female/male	79/76	135/105	0.304
Age, median (range)	78.4 (32.9–94.9)	80.3 (23.1–96.4)	0.666
Number of prescribed drugs, median (range)			
• Regular	9 (4–19)	8 (4–19)	0.107
• As needed	2 (0–10)	2 (0–11)	0.174
Living at home before admittance, n (%)	146 (94)	218 (91)	0.225
Assistance with drug administration:			
• Nursing home, n (%)	9 (6)	22 (9)	0.225
• Multidose, n (%)	46 (30)	49 (20)	0.035
• Home care, n (%)	30 (19)	31 (13)	0.084
Charlson Comorbidity Index, median score (range)	3 (0–11)	3 (0–11)	0.189
mean score (SD)	2.77 (1.97)	3.12 (2.16)	
Body-mass index ^b^, median (range)	23.8 (14.4–48.4)	25.0 (13.1–43.0)	0.145
Glomerular filtration rate (ml/min), median (range)	49.0 (9–182)	52.5 (5–235)	0.268
CYP2D6 poor metabolizers ^c^, n (%)	8 (5)	20 (9)	0.214
CYP2C19 poor metabolizers ^c^, n (%)	4 (3)	11 (5)	0.289
CYP2C9 *3 carriers ^c^, n (%)	18 (12)	23 (10)	0.569
SLCO1B1 *5 carriers ^c^, n (%)	38 (25)	66 (29)	0.472

^a^ Nine of the included patients were excluded from the comparison since defining hospitalizations as drug-related or not was impossible.

^b^ Body-mass index was registered for 121/155 patients with DRHs and 175/240 patients with non-DRHs.

^c^ Blood samples for genotyping were available for 150 patients (SLCO1B1) and 151 patients (cytochrome P450 (CYP)-enzymes) of 155 patients with DRHs and 230/240 patients with non-DRH

Table 2 shows comparisons between patients with DRHs and non-DRHs regarding the occurrence of DRPs. Patients with DRHs had significantly more DRPs in total, and significantly more of the DRP subgroups ‘drug monitoring’, ‘adverse effect’, ‘other’, ‘non-optimal drug therapy’, ‘reduced organ function / contraindication’ and ‘drug-drug interaction’. ‘Adherence issues’ were also observed more frequently in patients with DRHs versus non-DRHs (p = 0.050), and included in the multiple logistic regression analysis as a DRP subgroup.

**Table 2:** Overview of drug-related problems (DRPs) at hospitalization, identified by clinical pharmacists, in patients with drug-related hospitalization (DRH) versus non-drug-related hospitalizations (non-DRHs) in the study population (n = 395^a^).

DRPs	DRHs (155 patients)	Non-DRHs (240 patients)	p value
*Total*			
• Number of patients (%)	155 (100)	240 (100)	
• Number per patient, median (range)	15 (4–42)	12 (3–30)	<0.001
Subgroups			
*Drug monitoring*			
• Number of patients (%)	63 (41)	61 (25)	0.001
• Number per patient, median (range)	0 (0–4)	0 (0–2)	0.001
*Adverse effect*			
• Number of patients (%)	147 (95)	203 (85)	0.002
• Number per patient, median (range)	3 (0–10)	2 (0–8)	<0.001
*Non-optimal drug therapy*			
• Number of patients (%)	152 (98)	236 (98)	1.000
• Number per patient, median (range)	4 (0–10)	4 (0–11)	0.006
*Reduced organ function / contraindication*			
• Number of patients (%)	87 (56)	114 (48)	0.094
• Number per patient, median (range)	1 (0–8)	0 (0–7)	0.031
*Adherence issue*			
• Number of patients (%)	13 (8)	9 (4)	0.050
• Number per patient, median (range)	0 (0–2)	0 (0–2)	0.051
*Drug-drug-interaction*			
• Number of patients (%)	117 (76)	180 (75)	0.913
• Number per patient, median (range)	2 (0–10)	1 (0–10)	0.047
*Inappropriate drug in elderly*			
• Number of patients (%)	90 (58)	136 (57)	0.784
• Number per patient, median (range)	1 (0–6)	1 (0–6)	0.446
*Unnecessary drug*			
• Number of patients (%)	114 (74)	181 (75)	0.677
• Number per patient, median (range)	1 (0–7)	1 (0–7)	0.627
*Course length*			
• Number of patients (%)	57 (37)	93 (39)	0.693
• Number per patient, median (range)	0 (0–5)	0 (0–7)	0.921
*Practical problem*			
• Number of patients (%)	22 (14)	45 (19)	0.239
• Number per patient, median (range)	0 (0–2)	0 (0–4)	0.240
*Other*			
• Number of patients (%)	57 (37)	64 (27)	0.033
• Number per patient, median (range)	0 (0–4)	0 (0–5)	0.021

^a^ Nine of the included patients were excluded from the comparison since defining hospitalizations as drug-related or not was impossible.

Drug monitoring; Need for therapeutic drug monitoring. Adverse effect; Presence of symptoms or changes in laboratory values possibly caused by drug(s). Non-optimal drug therapy; Lack of drug treatment or non-optimal drug treatment of a symptom/disease. Course length; Consideration of appropriate duration of course length. Other; DRPs not applicable in other subgroups, e.g. prescription errors, documentation errors. The rest of the DRP subgroups are described in S1 Table.

In addition to the comparisons presented in Tables 1 and 2, it was observed that patients with multidose-dispensed drugs had significantly more DRPs in total than other patients (median number 15 (range 4–34) versus 14 (range 3–44); p = 0.038).

Table 3 shows adjusted OR with 95% CIs for the variables statistically significantly associated with DRHs, comprising occurrence of adverse effect DRPs (adjusted odds ratio (OR) 3.3, 95% confidence interval (CI) 1.4–8.0), adherence issues (OR 2.9, 1.1–7.2), home care (OR 1.9, 1.1–3.5), drug monitoring DRPs (OR 1.9, 1.2–3.0), and CCI score ≥6 (OR 0.33, 0.14–0.77).

**Table 3:** Adjusted odds ratios (OR) with 95% confidence intervals (CI) for the characteristics related to drug-related hospitalizations estimated in multiple logistic regression analysis.

Characteristic	Adjusted OR (95% CI)	p value
Adverse effect DRPs^a^	3.29 (1.36–7.99)	0.008
Adherence issue	2.86 (1.14–7.17)	0.025
Home care	1.93 (1.07–3.50)	0.030
Drug monitoring DRPs^a^	1.91 (1.21–3.00)	0.005
Charlson Comorbidity Index Score		
Score 0–1 (ref)	1	0.033
Score 2	0.70 (0.38–1.29)	0.249
Score 3	1.17 (0.63–2.16)	0.621
Score 4	0.55 (0.27–1.13)	0.102
Score 5	1.21 (0.50–2.91)	0.670
Score ≥6	0.33 (0.14–0.77)	0.011

^a^ drug-related problem

## Discussion

In this study on multimorbid internal medicine patients, almost 40% of hospitalizations were assessed as *possibly* being drug-related. To the best of our knowledge, the study is the first to characterize drug-related hospitalizations in multimorbid patients. The frequency of drug-related hospitalizations was very high and substantially higher than in many other patient populations. Drug groups associated with DRHs in previous studies [7, 11, 13], e.g. beta-blockers and diuretics, were similarly frequently involved in DRHs in the present study. Important considerations are that morbidity increases the vulnerability towards drug-related problems [36–38] and that the included patients used a minimum of four regular drugs, which generally increases the risk of DRPs [17, 39]. These results clearly indicate the necessity of managing DRPs in multimorbid patients.

Presence of three specific DRP subgroups was associated with significantly higher odds for DRHs among the included patients. Patients with suspected adverse effects and adherence issues had around a threefold increased odds of DRHs, and patients with drug monitoring DRPs had nearly a twofold increased odds. Similar findings have been reported in previous studies investigating the type of DRPs most frequently associated with DRHs in various patient populations [1–3, 6, 7]. However, the present study is the first to include specific DRP subgroups together with non-drug-related factors in a multiple logistic regression analysis to investigate the impact on risks of DRHs. By adjusting for different non-drug-related factors, adherence issues and adverse effects DRPs were identified as major risk factors for DRHs in this population of multimorbid internal medicine patients.

Patients with the highest CCI score had significantly reduced odds for DRHs compared to the reference group with the lowest CCI scores. This is an interesting finding as several other studies on other patient populations have found the opposite, i.e. that high comorbidity is associated with increased risk of being hospitalized due to drug-related issues [11, 40, 41]. The result from our study probably reflects that complex multimorbid patients are frequently hospitalized due to disease progression and that a high CCI score in the current study population indicates the presence of such an extensive disease/symptom complexity, which more often resulted in classifying a hospitalization as disease-related rather than possibly drug-related. The finding suggests that focus should be on optimizing drug treatment in the healthiest of the multimorbid patients, which is in accordance with the results of a previous Swedish study, where a pharmacist intervention was more effective in preventing emergency department visits in patients using fewer drugs [42]. However, additional studies are required to evaluate which patients will benefit the most from medication reviews to prevent DRHs.

Home care was associated with increased risk of DRHs in the study population. Immediately this appears as unexpected and provoking findings, but it is important to specify that patients requiring home care assistance to drug administration usually have increased frailty and impaired cognition. Thus, despite that the statistical analysis accounted for differences in CCI scores and identified DRPs, it might be that the statistically significant associations of home care to DRHs are confounded by factors related to the patient vulnerability that not were measured and included in the multivariate model, e.g. impaired cognition. On the other hand, it could not be excluded that drug management by the home care service is of such low quality that it increases the risk for DRHs. Actually, the latter is to some extent supported by reported findings in previous studies [43–45], which possibly may reflect insufficient cooperation between different health care providers [46, 47].

In contrast to several other studies [7, 11, 27], we did not find that the overall number of regularly used drugs was associated with an increased risk of DRHs. This might have been different if the study had investigated patient readmissions shortly after hospital discharge, as the 30-day readmission rate is in previous studies closely related to the number of drugs [48–50]. However, in the context of multimorbid patients, it appears that reducing the number of drugs per se is not a sufficient action to prevent DRHs and that closer follow-up of specific DRP subgroups, i.e. suspected adverse effects, adherence issues and drug monitoring, may potentially be rational.

A unique feature of the present study was that we investigated pharmacogenetic factors related to hospitalizations. While the frequencies of genotype-predicted poor metabolizers (PMs) did not differ between patients with and without DRHs, GDIs of potential clinical relevance were identified in the majority of the patients and considered to influence about 25% of the hospitalizations assessed as possibly drug-related. A drug often involved in the identified GDIs was metoprolol, which is mainly metabolized by the enzyme CYP2D6. In white Europeans, 5–10% are CYP2D6 PMs. These patients obtain a five-fold higher dose exposure and are therefore at substantially increased risk of excessive beta-blockade and accompanying consequences like hypotension, dizziness, and falls. This is the main reason why metoprolol was frequently linked to DRHs, but an important point is also that the use of metoprolol likely prevents many hospitalizations as well [51, 52]. Nevertheless, the frequent occurrence of clinically relevant GDIs in the study population suggests that pharmacogenetics should be included as an aspect in future studies investigating risk factors for DRHs.

We did not consider the preventability of the DRHs in the study, which represents an important limitation. However, the significantly higher frequencies of several DRP subgroups, which generally are regarded as preventable (i.e. adverse effects, adherence issues and drug monitoring), in patients with DRHs versus non-DRHs indicates that a major proportion of the cases assessed as DRHs could have been avoided.

In multimorbid patients, symptoms and disease states at hospital admission are complex, which limits the suitability of objective scoring tools as a basis for assessing relationships between drug use and hospitalizations. Thus, we decided to use a method for individual and comprehensive assessments using complete sets of clinical and pharmacological data to classify hospitalization as ‘possibly’ or ‘unlikely’ drug-related. This is a time-consuming method but enables detailed assessments of clinical characteristics related to the admissions. On the other hand, the validity of the methodological approach is difficult to evaluate. The occurrence of adherence issues, adverse effect, and drug monitoring DRPs were found associated with increased risk of DRHs as in other studies involving older patients, which supports the validity of the DRH assessments in the present study.

It is likely that the findings of our study will reflect other multimorbid patient populations using multiple drugs from different therapeutic classes. However, as for other studies investigating DRHs, the generalisability of the findings are not necessarily transferable to other hospitals or clinical setting. Thus, further studies on the same patient group, preferably also including additional factors of potential importance for the risk of hospitalization, e.g. social factors, should be conducted to compare and evaluate the generalisability of our findings.

Important limitations of the study include i) the lack of measuring inter-rater reliability between the six clinical pharmacists involved in the identification and classification of DRPs, ii) the lack of using of an internationally agreed classification system for DRPs (IMM classification used instead), iii) the lack of assessing preventability of DRHs and iv) that the DRH assessments were performed by only two researchers/experts without applying a validated scale. However, regarding the first mentioned limitation, a point is that the involvement of several clinical pharmacists also is a study strength, as it also may reduce the probability of operator-biased findings. The thorough, comprehensive DRH assessments covering all aspects related to the hospitalizations is another strength of the study.

## Conclusions

This study indicates that DRHs are prevalent in multimorbid internal medicine patients using at least four regular drugs from different therapeutic classes. Several factors were associated with risk of DRHs in these patients, with adverse effect DRPs and adherence issues being most important. The results clearly indicate the necessity of managing DRPs in multimorbid patients.

## Supporting information

S1 Table Detailed description of the subgroups of drug-related problems (DRPs). (PDF)

S2 Table Overview of the variant alleles (mutations) of the various pharmacogenes included in the genotyping panels and the respective genotype-predicted aberrant phenotypes required for gene-drug interaction (GDI) assessments. (PDF)

S3 Table Overview over the number of times a drug was suspected to be related to hospitalization in 155 drug-related hospitalizations, ranked according to frequency (drugs associated 1 or 2 times are not included in the overview). (PDF)

S4 Table Overview of gene-drug interactions (GDIs) identified during the retrospective reviews of the reconciled drug lists in relation to the respective patients’ genotype results. GDIs of potential relevance for assessments of drug-related hospitalizations (DRHs) were determined based on expected consequences of GDIs and information available in the medical records, including causes of hospitalization.
(PDF)
