# Supplementary material for: Prevalence and risk factors of drug-related hospitalizations in multimorbid patients admitted to an internal medicine ward
Source: PLoS One. 2019 Jul 22;14(7):e0220071. doi: 10.1371/journal.pone.0220071 (PMC6645516; doi:10.1371/journal.pone.0220071)
Supplement: S1 Table — (PDF) [file pone.0220071.s001.pdf]

S1 Table. Detailed description of the subgroups of drug-related problems (DRPs).

DRP subgroup	Detailed description
Drug monitoring	Need for therapeutic drug monitoring
Adverse effects	Presence of symptoms or changes in laboratory values possibly caused by drug(s)
Drug-drug interaction	Clinically relevant drug-drug interactions
Non-optimal drug therapy	Lack of drug treatment or non-optimal drug treatment of a symptom/disease
Reduced organ function / contraindication	Drug or dosage of drug inappropriate due to reduced kidney function, reduced liver function, contraindications or other diseases.
Inappropriate drug in elderly	Use of less favourable drugs in patients over 65 years old, e.g. anticholinergics
Unnecessary drug	Drug in use is not indicated
Course length	Consideration of appropriate duration of course length, e.g. duration of antibiotics
Practical problem	Practical challenges in drug handling, e.g. inhalation devices
Adherence issue	Patient do not, intentional or unintentional, use / take drug as agreed
Other	DRPs not applicable in other subgroups, e.g. prescription errors, documentation errors
