# Supplementary material for: Prevalence and risk factors of drug-related hospitalizations in multimorbid patients admitted to an internal medicine ward
Source: PLoS One. 2019 Jul 22;14(7):e0220071. doi: 10.1371/journal.pone.0220071 (PMC6645516; doi:10.1371/journal.pone.0220071)
Supplement: S2 Table — (PDF) [file pone.0220071.s002.pdf]

S2 Table. Overview of the variant alleles (mutations) of the various pharmacogenes included in the genotyping panels and the respective genotype-predicted aberrant phenotypes required for gene-drug interaction (GDI) assessments.

Gene	Variant allele panel	Genotype-predicted aberrant phenotypes
CYP2D6 [1]	Non-coding variant alleles:<br>• 2D6*3 (rs35742686)<br>• 2D6*4 (rs3892097)<br>• 2D6*6 (rs5030655)<br>Reduced-function variant alleles:<br>• 2D6*9 (rs5030656)<br>• 2D6*10 (rs1065852)<br>• 2D6*41 (rs29001518)<br>Copy number analysis:<br>• 2D6*5 (whole gene deletion)<br>• multiplication of fully-functional alleles (2D6*1 or *2)	'Poor metabolizers' (PMs): Homozygous carriers of non-coding alleles<br><br>'Intermediate metabolizers' (IMs): Heterozygous carriers of non-coding alleles and homozygous carriers of reduced-function alleles<br><br>'Ultrarapid metabolizers' (UMs): Carriers of three or more functional gene copies
CYP2C19 [1]	Non-coding variant alleles:<br>• 2C19*2 (rs4244285)<br>• 2C19*3 (rs4986893/rs57081121)<br>• 2C19*4 (rs28399504)<br>Gain-of-function allele:<br>• CYP2C19*17 (rs12248560)	PMs: Homozygous carriers of non-coding alleles<br><br>IMs: Heterozygous carriers of non-coding alleles<br><br>UMs: Hetero- or homozygous carriers of the gain-of-function allele
CYP2C9 [1]	Reduced-function alleles:<br>• 2C9*2 (rs1799853)<br>• 2C9*3 (rs1057910)	PMs: Homozygous carriers of CYP2C9*3<br><br>IMs: Heterozygous carriers of CYP2C9*3 and homozygous carriers of CYP2C9*2
CYP3A5 [1]	Non-coding variant allele:<br>• CYP3A5*3 (rs776746)	Increased CYP3A5 metabolism: Hetero- or homozygous carriers of CYP3A5*1
SLCO1B1 [2]	Reduced-function allele:<br>• 521T>C/*5 (rs4149056)	Decreased OATP1B1-mediated transport, e.g. of statins: Hetero- or homozygous carriers of the reduced-function allele
VCORK1 [3]	Increased VCORK1-sensitivity:<br>• VKORC1*2 (rs9923231)	Low-dose warfarin responders:<br>Hetero- or homozygous carriers of VKORC1*2

All genotyping assays were validated and had been certified by Norwegian accreditation for routine clinical use. All variant allele analyses were performed using Taqman-based realtime PCR assays.

REFERENCES:

[1] Gaedigk A, Ingelman-Sundberg M, Miller NA, Leeder JS, Whirl-Carrillo M, Klein TE. The Pharmacogene Variation (PharmVar) Consortium: Incorporation of the Human Cytochrome P450 (CYP) Allele Nomenclature Database. Clin Pharmacol Ther. 2018; 103: 399-401.

[2] Kalliokoski A, Niemi M. Impact of OATP transporters on pharmacokinetics. British journal of pharmacology. 2009; 158: 693-705.

[3] Owen RP, Gong L, Sagreiya H, Klein TE, Altman RB. VKORC1 pharmacogenomics summary. Pharmacogenetics and genomics. 2010; 20: 642-4.
