# Supplementary material for: Prevalence and risk factors of drug-related hospitalizations in multimorbid patients admitted to an internal medicine ward
Source: PLoS One. 2019 Jul 22;14(7):e0220071. doi: 10.1371/journal.pone.0220071 (PMC6645516; doi:10.1371/journal.pone.0220071)
Supplement: S3 Table — (PDF) [file pone.0220071.s003.pdf]

S3 Table. Overview over the number of times a drug was suspected to be related to hospitalization in 155 drug-related hospitalizations, ranked according to frequency (drugs associated 1 or 2 times are not included in the overview).

**Drugs**	**Suspected relation with hospitalization, n**
Metoprolol	39
Bumetanide	21
Zopiclone	15
Insulin and insulin analogues	13
Acetylsalicylic acid<br>Furosemide	11
Oxazepam<br>Warfarin	10
Atorvastatin	9
Enalapril<br>Oxycodone<br>Simvastatin	8
Buprenorphine<br>Formoterol/budesonide<br>Escitalopram	7
Codeine/paracetamol<br>Ipratropium bromide<br>Isosorbide mononitrate<br>Losartan<br>Tramadol	6
Amlodipine<br>Bisoprolol<br>Glimepiride<br>Glyceryl trinitrate<br>Nifedipine<br>Paracetamol<br>Prednisolone<br>Ramipril<br>Salbutamol	5
Calcium, combinations with vitamin D and/or other drugs<br>Citalopram<br>Digoxin<br>Lisinopril<br>Metformin<br>Methotrexate<br>Nitrazepam<br>Pantoprazole<br>Rivaroxaban<br>Zolpidem	4
Chlorprothixene<br>Cortisone<br>Hydrochlorothiazide<br>Levothyroxine sodium<br>Mycophenolic acid<br>Solifenacin<br>Spironolactone	3
