# Supplementary material for: Prevalence and risk factors of drug-related hospitalizations in multimorbid patients admitted to an internal medicine ward
Source: PLoS One. 2019 Jul 22;14(7):e0220071. doi: 10.1371/journal.pone.0220071 (PMC6645516; doi:10.1371/journal.pone.0220071)
Supplement: S4 Table — GDIs of potential relevance for assessments of drug-related hospitalizations (DRHs) were determined based on expected consequences of GDIs and information available in the medical records, including causes of hospitalization. (PDF) [file pone.0220071.s004.pdf]

S4 Table. Overview of gene-drug interactions (GDIs) identified during the retrospective reviews of the reconciled drug lists in relation to the respective patients' genotype results. GDIs of potential relevance for assessments of drug-related hospitalizations (DRHs) were determined based on expected consequences of GDIs and information available in the medical records, including causes of hospitalization.

Gene	Drug (*=prodrugs)	Total GDIs, n	Relevant for DRH assessments, n
CYP2C9	Warfarin	19	2
	Losartan*	10	1
	Ibuprofen	5	
	Candesartan	4	
	Irbesartan	3	
	Fluvastatin	2	
	Diclofenac	2	
	Glimepiride	1	
CYP2C19	Pantoprazole	58	2
	Esomeprazole	20	2
	Clopidogrel*	16	1
	Diazepam	11	
	Escitalopram	10	1
	Citalopram	5	1
	Omeprazole	3	
	Lansoprazole	3	
	Doxepin	1	
	Fluoxetine	1	1
	Sertraline	1	
	Venlafaxine	1	1
CYP2D6	Metoprolol	125	15
	Codeine*	42	2
	Oxycodone	19	1
	Tramadol*	11	3
	Amitriptyline	6	
	Mirtazapine	5	
	Haloperidol	3	
	Carvedilol	2	
	Simvastatin	2	2
	Fesoterodine	2	
	Chlorprothixene	2	2
	Risperidone	2	2
	Mianserin	2	
	Tamsulosin	2	1
	Levomepromazine	1	
	Prochlorperazine	1	1
	Zuclopenthixol	1	1
	Venlafaxine	1	1
	Donepezil	1	
CYP3A5	Simvastatin	17	
	Atorvastatin	11	
	Apixaban	3	
	Amlodipine	3	
	Rivaroxaban	2	
	Clopidogrel*	1	
	Nifedipine	1	
	Pravastatin	1	
	Vardenafil	1	

	Fentanyl<br>Buprenorphine<br>Zopiclone	1<br>1<br>1	
SLCO1B1	Simvastatin<br>Atorvastatin<br>Pravastatin<br>Fluvastatin	23<br>21<br>2<br>2	1<br>4
VCORK	Warfarin	41	4
TOTAL		538	52

CYP = cytochrome P450
